# Porous Metals: Preparation, Microstructure, Properties and Performance

**DOI:** 10.3390/ma18245518

**Published:** 2025-12-08

**Authors:** Yuyuan Zhao

**Affiliations:** School of Mechanical and Automotive Engineering, Ningbo University of Technology, Ningbo 315211, China; zhaoyuyuan@nbut.edu.cn

Porous metals are a special class of composite materials, consisting of a metal phase and a gaseous phase. Porous metals are also known as metal foams and cellular metals, although these have different connotations. Porous metal is a general term referring to any solid metal containing internal pores, no matter whether they are closed or open, or regularly or irregularly distributed. Metal foam is a type of porous metal manufactured by a foaming process, either by injecting gas into or generating gas in the liquid metal, and generally has closed pores. Cellular metal emphasizes the cell-based pore structure, often with a regular pattern. In practice, however, the boundaries between these terms are blurred and they are often used interchangeably.

Rapid developments have been made in the field of porous metals in the past three decades. A number of introductory books and review articles on the manufacture, properties, and applications are available in the literature [1,2,3,4,5,6,7,8,9,10,11,12,13,14].

The functionality of porous metals is derived from the combinations of two distinct materials, metal and air. Porous metals are finding applications in many sectors, such as aerospace, automotive, construction, and energy, due to their unique properties. The applications of porous metals can largely be categorized into two classes: mechanical energy absorption due to their ability for a large amount of plastic deformation, and non-mechanical functions such as filtering, heat dissipation, electromagnetic shielding, sound absorption, etc. Many studies have consequently been conducted on mechanical [7,8,9,12,15,16,17,18,19] and non-mechanical [20,21,22,23,24,25,26,27,28,29,30,31] properties. The properties of porous metals are not only dependent upon the metal matrix, but are particularly sensitive to the nature of the porous structure, characterized by the shape, size, quantity, and connectivity of the pores.

The porous structure is closely related to the technology used to produce the porous metals. A variety of techniques have been used to manufacture porous metals, including foaming, casting, sintering, and 3D printing [6,10,11,13,14,32,33,34,35,36,37,38,39]. A useful way to understand the performance of porous metals produced by these different technologies is to look into the specific as-produced porous structures and to examine how the structures affect their properties.

This Special Issue, “Porous Metals: Preparation, Microstructure, Properties and Performance”, presents a collection of ten publications. They give a snapshot of recent advances in the manufacturing and characterization of porous metals. They cover several typical metals and their alloys, including Al [40,41,42], Cu [43], Ti [44,45,46], Fe-Ni [47], and metal–organic framework (MOF) [48]. A range of manufacturing methods have been employed, including foaming [40,42], sintering [44,45,46], space holder [41,43,49], template electroplating [47], and hydrothermal synthesis [48]. While most studies [40,42,43,45,46,47] focus on mechanical properties, non-mechanical properties, such as surface area [48], capillary effect [41], and fluid flow and heat transfer [49], are also investigated.

This Editorial summarizes the main findings reported in the ten publications in this Special Issue. It is not intended to give comprehensive coverage of all the key points. Instead, only the special or unique features, which may be of particular interest to the readers, are given an introduction or discussion.

Kertesz & Kovacs [40] investigated the energy-absorbing capacity of a closed-cell aluminum foam manufactured by a foaming process using Al powder as the raw material and TiH_2_ as the foaming agent. The main aim of the investigation is to study the role played by friction caused by the confining tube wall, through free and constrained compression tests. They found that confinement inhibited cell deformation and friction between the foam and the tube wall consumed about 20% of the compressive energy. Chu et al. [42] tackled the low-thermal conductivity problem of Al foams manufactured via foaming processes. Poor thermal conductivity leads to large-sized Al alloy foams being difficult to be homogeneously strengthened by long-time age hardening. They adopted the Al-0.16Sc-0.17Zr alloy and demonstrated that this alloy can be subjected to prolonged aging because the Al_3_(Sc, Zr, Ti) phase has a core–shell structure and the precipitates are less prone to coarsening.

Olmos et al. [45] manufactured porous Ti6Al4V materials by extrusion 3D printing plus sintering. They produced precursor filaments by extrusion of Ti6Al4V powder with polymer additives and printed samples with open channels by filament building up, followed by sintering. The as-produced porous structures have two types of porosity: the open channels and the fine inter-particle porosity in the struts. The two-scaled porosity is considered beneficial to bone implant applications. Villa-Tapia et al. [46] manufactured porous Ti6Al4V alloy compacts by sintering first and then infiltrated liquid Ag into the compacts to produce Ti6Al4V/Ag composites, which have potential as antibacterial materials for biomedical use. Yang et al. [44] manufactured porous Ti by sintering Ti powder in a molten MgCl_2_/Mg bath and reported that Mg promoted the aggregation and densification of the Ti particles, due to good wettability between Mg and Ti, and enhanced precipitation of dissolved titanium atoms from molten Mg.

Xiao et al. [43] investigated the mechanical properties of porous Cu manufactured by the space holder method. They studied two types of porous structures, created by two space holder particle shapes, with a wide porosity range. They confirmed that pore shape has a significant effect on the mechanical properties. Although the elastic modulus and yield strength follow the empirical power law with relative density, different pore shapes give different power law constants. Shen et al. [41] studied the capillary effect of porous aluminum wicks manufactured by the space holder method. They created a bi-porous structure using two different-sized NaCl powders. They showed that an optimized combination of large and small pores can generate a higher capillary pressure than the mono-porous structure with the same porosity. Lu et al. [49] conducted a numerical study on fluid flow and heat transfer of porous media manufactured by the space holder method. They investigated the effects of pore size, porosity, and flow velocity on the pressure drop, permeability, form drag coefficient, and heat transfer coefficient, and showed good agreements with existing experimental results. It is worth noting that their results showed that, at high velocity, only the metal foam close to the heat source contributes to heat dissipation. It is a good example of showing the benefits of numerical modeling in saving resources. Both studies [41,49] provided some useful information for heat exchanger applications of this type of porous metal.

Zhang et al. [47] investigated the energy absorption characteristics of fiber–foam sandwich structures subjected to gas explosion. The sandwich structures consisted of iron–nickel foam being used as the front and rear panels, and fiber materials (carbon, aramid, and glass) functioning as the core layer. They reported that the combination of rigid metal foam plates and a flexible fiber layer improved the energy absorption performance. However, no quantitative information on their relative contributions is elaborated.

Zhao [48] manufactured nanofused MOFs based on copper and trimesate through a low-temperature hydrothermal method. The as-fabricated MOFs have specific surface area (BET) as high as 1220 m^2^·g^−1^, and demonstrated markedly improved catalytic performance in the cyanosilylation of aldehydes.

In reviewing these papers in this Special Issue, it is easy to conclude that the scope of research in the field of porous metals is constantly expanding. The porous metals community constantly endeavor to develop new manufacturing technologies, produce new materials, create new structures, identify new properties, and find new applications. The field has many exciting research topics to offer.
